# Comparison of Overpressured-Layer Chromatography and High-Performance/High-Pressure Layer Electrochromatography Using the New Prototype Equipment in Various Operational Modes

**DOI:** 10.3390/molecules27134032

**Published:** 2022-06-23

**Authors:** Radosław Łukasz Gwarda, Tadeusz Henryk Dzido

**Affiliations:** Department of Physical Chemistry, Faculty of Pharmacy, Medical University of Lublin, 4a Chodźki Str., 20-093 Lublin, Poland; tadeusz.dzido@umlub.pl

**Keywords:** high-performance (high-pressure) layer electrochromatography, overpressured-layer chromatography, thin-layer chromatography, high-throughput separation, forced-flow chromatography, planar separation, separation techniques comparison, multichannel separation, simultaneous separation, dye separation

## Abstract

In our previous paper we have presented the new prototype equipment and introduced a new analytical technique—high-performance/high-pressure layer electrochromatography (HPLEC), a combination of overpressured-layer chromatography (OPLC) and pressurized planar electrochromatography (PPEC). In this paper, the work of the equipment in various operational modes is investigated. Some difficulties and challenges related to various aspects of separation are discussed. The OPLC and HPLEC techniques are compared in terms of selectivity and performance. The results show that our equipment can be successfully used for singe- and multichannel OPLC and HPLEC separations in various sample application and detection modes. It includes the high-throughput, multichannel, and fully automated online separation of multiple samples simultaneously. The equipment allows for the independent optimization of various operational parameters. HPLEC combines the advantages of column/capillary and planar separation techniques while overcoming their limitations. It also combines the advantages and overcomes the drawbacks of OPLC and PPEC. It provides hydrodynamic flow of the mobile phase, irrespective of the voltage used and/or the mobile phase composition. Thus, any optimization of the composition and the voltage can be used independently. Both can be used to obtain the required selectivity of separation. The voltage can be used to facilitate the mobile phase flow and accelerate the analysis.

## 1. Introduction

In our previous paper [1], we have presented the new prototype equipment for the new analytical technique—high-performance/high-pressure layer electrochromatography (HPLEC). The technique is a combination of overpressured-layer chromatography (OPLC) and pressurized planar electrochromatography (PPEC). It combines their advantages while overcoming their limitations. The technique provides fast and effective analysis performed in a fully closed separation system. It is obtained by forcing the rapid and uniform flow of the mobile phase through the adsorbent layer, using a high-pressure pump. HPLEC enables the simultaneous analysis of multiple samples. It provides the possibility of an easy change of separation selectivity, by the addition of the electrophoretic effect to the overall mechanism of separation. Contrary to PPEC, HPLEC enables one to obtain the flow of the mobile phase that does not depend on the voltage used and the electroosmotic effect induced. Thus, the voltage and the mobile phase composition may be optimized independently. Contrary to OPLC, besides the change of separation selectivity mentioned, it can also provide the additional enhancement of the mobile phase flow and the reduction of the backpressure. This may be used to further speed-up of the analysis. Moreover, the addition of the electroosmotic effect to the overall mechanism of the mobile phase flow can be supposed to change the flow profile (from laminar to flat) and increase the efficiency of the separation system. Besides those clear advantages of HPLEC, the equipment present in our latest paper still can be used to perform solely OPLC or PPEC.

The HPLEC equipment was designed to work in various operational modes, concerning the number of samples analyzed simultaneously, the mobile phase flow ratio: flow from the main pump vs. flow from the sample pump, and the sample application and detection mode (online, offline, or their combination). In our previous paper, we have shown only some very preliminary results concerning the fully online and fully automated, simultaneous, multichannel HPLEC separation of analgesic drugs in a reversed-phase system [1]. The others operational modes were not investigated yet.

Various separation modes have been reported for PPEC and OPLC. The most PPEC analysis was performed with an offline sample application and offline detection. Only two papers presented PPEC with an online sample application [2,3], but no online detection was reported. All of the PPEC analyses were performed with water-containing mobile phases—either in a normal- or a reversed-phase separation system [4,5,6,7,8,9].

Most of the reported OPLC analyses were performed in normal-phase systems, with the use of the organic (nonpolar) mobile phases. Some of the reversed-phase OPLC separations were also described. Both offline and online sample application and detection (as well as their combinations) have been presented [6,7,10,11,12,13,14]. The majority of papers concern the offline sample application mode as online sample injection resulted in relatively low efficiency of separation (due to dispersion of solute starting zone) [15]. With the online sample detection mode, mainly the single-channel analysis was performed, but two-channel experiments were also reported [13].

The aim of this work is the demonstration that our new prototype HPLEC equipment is able to work in many different operational modes, as has been assumed during its design and construction. It also aims to discuss the difficulties and challenges related to using the equipment in general, as well as with individual operational modes. Finally, it features a comparison and discussion of the results obtained with OPLC and HPLEC in similar separation systems.

## 2. Results and Discussion

**Figure 1:**
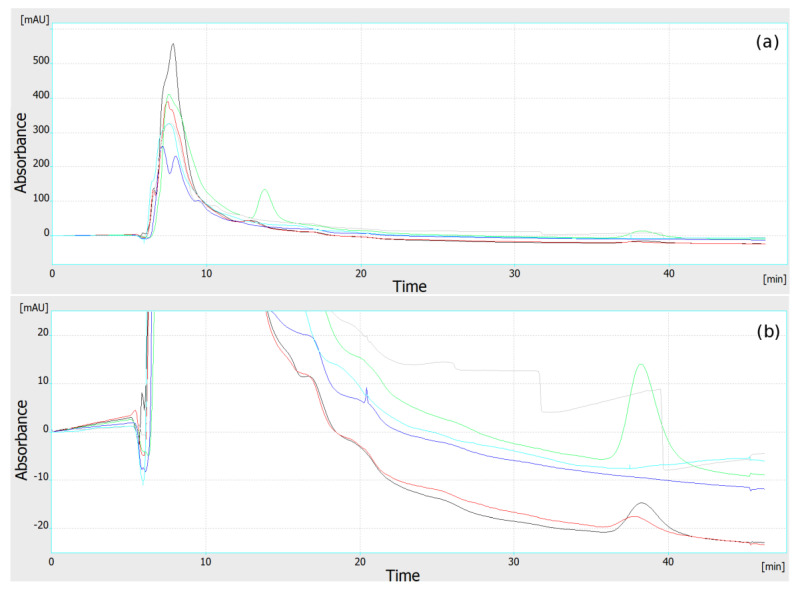
Preconditioning (equilibration) of separation system. Six chromatograms (each of different color) from six independent detectors obtained simultaneously, during procedure of adsorbent layer washing: (**a**) full view, (**b**) scaled down fragment. All detectors respond at the same time to the front of the mobile phase flow. Baselines do not reach plateau for over 45 min of equilibration. Mobile phase flow 0.25 mL/min.

Papers concerning OPLC describe some problems with the use of unequilibrated separation systems, which are related to multiple and/or jagged fronts of the mobile phase [14,16,17,18,19]. Our results show that long washing/preconditioning of the adsorbent layer with the mobile phase is needed to obtain a flat and smooth baseline (Figure 1), and to obtain reliable, repeatable results. This is independent of the backpressure, which reaches its working value (here about 80–95 bar) and plateau in about 10–15 min. With the conditions used in our experiments (especially flow velocity of the mobile phase about 0.25 mL/min), 1.0–1.5 h of adsorbent preconditioning was needed before analysis, to obtain a flat baseline and the reliable response of the detectors. In any case, our preliminary observations suggest that after the equilibrium is finally obtained, the single chromatographic plate can be used over 24 h, without any noticeable change of selectivity (still, this issue should be further investigated in detail). The need for such long preconditioning probably results mainly from the presence of impurities in the adsorbent layer. As we have shown elsewhere [20], HPTLC plates may contain a considerable number of various metal cations. These may originate from the adsorbent binder, adsorbent alone, and/or may be some accidental impurities. On the other hand, the baseline drift may also originate from progressive removal of the air from the pores of the adsorbent by its dissolution in the flowing mobile phase [14]. It must be mentioned that the preconditioning of adsorbent layer changes its properties and thus the selectivity of the separation system. Some of our initial results proved that using the same adsorbent and the same mobile phase, retention of azo dyes is much lower in OPLC, in comparison to the HPTLC system (results not shown/registered). This made it impossible to transfer optimized HPTLC conditions to the OPLC system (as the most of dyes separated in HPTLC were eluted near t0 in OPLC).

A similar problem appeared when we applied high voltage to the system. When we immediately used high voltage (about 4 kV), the electric current exceeded the capabilities of the power supply (electric current 150 mA, power 300 W). Moreover, the equipment cooling system was not efficient enough to balance the Joule’s heat and to maintain the separation temperature set. Therefore, it was necessary, to use a voltage gradient over long time, to precondition the adsorbent layer with high voltage. Here, we used a voltage gradient from 0 to about 4 kV (depending on analysis) for 1 h. Usually, we used a voltage gradient along with the preconditioning of the adsorbent with the mobile phase, to save the time. Using such a procedure made it possible to maintain an electric current about 50 mA and maintain effective cooling of the separation system. Moreover, it seems that using voltage during the preconditioning facilitated the process. That resulted in obtaining the flat baseline in a shorter time than without voltage (still this issue requires an additional, detailed investigation). These observations also support the explanation suggested above. By applying the voltage, ionic impurities are being removed from the adsorbent layer by the electrophoretic effect. After that, the separation system reaches its equilibrium, enabling one to use relatively high voltage with relatively low electric current and low heating. A similar observation and explanation were described before, for opened planar electrochromatography system [21].

Figure 1 shows that during the prewetting of the adsorbent layer, all 6 detectors responded approximately at the same time (with a maximum difference below 0.5 min). This suggests that the flow profile of the mobile phase through the adsorbent layer is smooth and uniform. This was confirmed by the multichannel test of the sample application and the detection described in our previous paper [1].

### 2.1. Single-Channel Fully Online Separation

**Figure 2:**
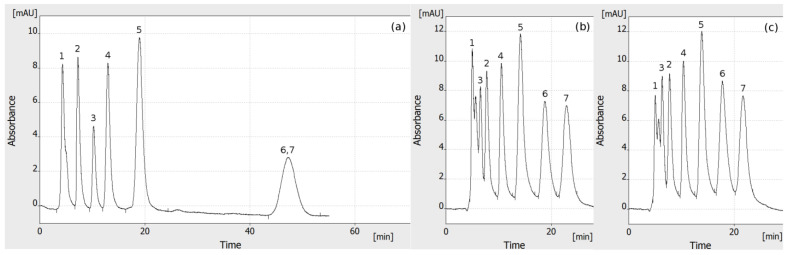
Comparison of single-channel (**a**) OPLC and (**b**,**c**) HPLEC of the dye mixture: 1 µL of the mixture was applied to the chromatographic plate (single channel), followed by the constant flow of the mobile phase from the sample pump. The separation system used was as follows: HPTLC RP-18 W chromatographic plates; water/methanol (3/2 *v/v*) with addition of 80 mM ammonium formate pH 3.0 as the mobile phase; total mobile phase flow 0.33 mL/min; voltage (**a**) 0 kV, (**b**) 3.7 kV, and (**c**) 3.8 kV; temperature 25 °C, cushion pressure 100 bar; and mobile phase backpressure (**a**) 93 bar, (**b**) 82 bar, and (**c**) 80 bar. Dye mixture: 1—indigotine 0.0125% *w/v*; 2—sunset yellow 0.025% *w/v*; 3—o-nitroaniline 0.0125% *w/v*; 4—allura red 0.0375% *w/v*; 5—azorubine 0.125% *w/v*; 6—1-aminoantraquionone 0.025% *w/v*; and 7—patent blue 0.125% *w/v*.

In the paper mentioned above, we have shown an exemplary HPLEC analysis of simple (two-component) analgesic drugs mixtures—Metafen and Poltram Combo Forte solutions [1]. Here, we present a comparison of OPLC (Figure 2a) and HPLEC (Figure 2b,c) of more complex dye mixture in similar separation systems. As our PPEC equipment offers various separation/operational modes, initially we have optimized the separation conditions using only a single separation channel and constant flow of the mobile phase from the sample pump. Both techniques used offered relatively good separation and satisfactory peak shape. Using the same flow of the mobile phase, the mixture was separated in about 50 min in OPLC system, while separation of the same mixture in the HPLEC system was about two times shorter. The separation selectivity in OPLC and HPLEC was different as the electrophoretic effect took part in the later. The elution sequence has changed in HPLEC (o-nitroaniline was eluted before sunset yellow; numbers 2 and 3 in Figure 2, respectively) in comparison to OPLC. Additionally, 1-aminoantraquionone and patent blue (no. 6 and 7 in Figure 2, respectively) were separated in HPLEC, while they coeluted in OPLC. An additional peak appears in HPLEC between peak no. 1 and 3. It probably origins from some impurity, which cannot be separated in OPLC. The position of this additional peak on the electrochromatogram deepens on the voltage used (compare Figure 2b,c). This means that even minor changes of voltage can result in changes of separation selectivity. As the separation time is reduced in HPLEC and the peaks are near to each other, the separation of their bases is somewhat worse. The results show a clear reduction of the HPLEC analysis time. However, the overall resolution of separation is reasonably decreased, despite the lowering of peak width at its half height (e.g., about 15% for peaks nos. 4 and 5).

The presented results proves that our equipment enables one to perform a satisfactory analysis in both the OPLC and HPLEC systems. In comparison to the most of OPLC results presented so far, these constitute a fully online analysis, performed with fully automated equipment. A single chromatographic plate can be used for multiple analysis. The subsequent analysis can be started and performed automatically, the same as in HPLC; there is no need for any operator intervention. The separation is performed in a fully equilibrated separation system, so the results are reliable and repeatable. The efficiency of separation (peak width) is acceptable. It seems that the main problem in this matter is the size of the solute starting zone. Other authors reported a similar problem concerning fully online OPLC experiments with offline sample application resulted in higher separation efficiency [15]. The optimization of online sample application is difficult, mainly due to technical reasons. These especially concern the presence of dead volumes and the mixing of various liquids in the sample application system. The “extra-column” broadening of the sample zone has a great share in the final separation efficiency. However, this problem was not investigated here, and the efficiency of separation was not optimized. It seems that it may depend to a significant extent on the sample application parameters (e.g., the ratio of the sample/mobile phase flow). Another problem may be the interactions of solutes with free silanols of the adsorbent layer—they may result in a mixed mechanism of retention and undesirable solute zone broadening [20,22,23,24]. The issue is deep and complex, it should be investigated in detail elsewhere.

While both OPLC and HPLEC analysis gave satisfactory preliminary results, it is clear that the later may show some advantages over the former. As it was proven above, the voltage can be used to change the separation selectivity, due to the addition of the electrophoretic effect. Probably in most cases, OPLC can be used and optimized as a base method, and then the voltage can be used to obtain additional changes of selectivity required, to perform further optimization of separation system. As even the minor changes to the applied voltage result in changes of separation selectivity, they can be used to obtain even subtle changes of peak position on electrochromatogram and thus, e.g., improve the separation of peak bases or separate some impurities from the main peak. Additionally, an effect can be used to decrease the backpressure during the analysis. Therefore, a higher flow of the mobile phase can be used, with the same pressure limit. The most important point, contrary to PPEC, is that in HPLEC the flow of the mobile phase, in general, does not depend on the electroosmotic effect. The mobile phase composition and properties can be optimized (e.g., pH, polarity) to obtain required retention, without paying attention to their influence on electroosmosis. This presents many possibilities to optimize various HPLEC parameters to obtain the best results expected, as discussed elsewhere [1].

### 2.2. Multichannel Fully Online Separation

As the advantages of HPLEC discussed above are clear, it is assumed that the true power of this technique is high throughput [1]. Therefore, the next step of our work was an attempt to perform multichannel analysis using similar separation systems. Here, a problem appeared, as the results obtained with sequential sample application, with only the temporary flow of the mobile phase from the sample pump, were different from these presented above. The problem resulted from the different ratio of the mobile phase flow from the main pump and the sample pump. This affected the general flow profile, as well as the shape of the sample zone. The zone shape of the injected sample is a complex issue, so it should be investigated in detail elsewhere. In the experiments presented above, in the general flow profile, the mobile phase flow in the separation channel originated mainly from the sample pump (as the mobile phase was pumped during the whole experiment after the injection of the sample). It was only supported and directed (by the means of the pressure distribution in the various areas of the adsorbent layer and the pressure drop) by the flow from the main pump. The linear flow in the separation channel was also higher than in the rest of the adsorbent layer (a shorter distance between the sample inlet and outlet than between mobile phase inlet and outlet, and thus lower backpressure). In multichannel separations, the overall flow of the mobile phase originated from the main pump and was uniform throughout the whole adsorbent layer (the same in all separation channels). For that reason, the operational parameters had to be optimized separately for single-channel (with continuous flow of the mobile phase from the sample pump) and multichannel separations. The differences between single- and multichannel separations were minor for OPLC but more numerous for the HPLEC experiments. This shows that the ratio of linear mobile phase flow vs. applied voltage is crucial for the selectivity of separation. In OPLC, the change of the mobile phase flow results only in a change of time of the analysis but not a change of separation selectivity. In HPLEC, a change of the flow results in a change of the time in which the analyte is under the influence of electric voltage. This, in turn, results in a change of the selectivity. Therefore, HPLEC separation conditions cannot be simply transferred between systems with continuous and discontinuous flow from the sample pump.

**Figure 3:**
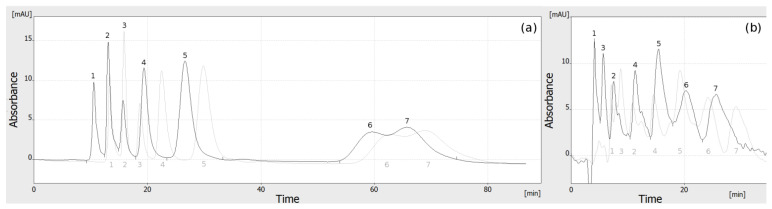
Comparison of multi-channel OPLC (**a**) and HPLEC (**b**) of the dye mixture: 1 µL of the mixture was applied to the five separation channels, with 3 min delay between sample application in each subsequent channel. Signals from only two channels are shown—no. III (black) and no. IV (grey). Separation system used: HPTLC RP-18 W chromatographic plates; water/methanol (3/2 *v/v*) with addition of 80 mM ammonium formate pH 3.0 as the mobile phase; total mobile phase flow 0.25 mL/min (**a**) 0.34 mL/min (**b**); voltage 0 kV (**a**), 4.2 kV (**b**); temperature 25 °C, cushion pressure 100 bar; and mobile phase backpressure 96 bar (**a**), 93 bar (**b**). Dye mixture: 1—indigotine 0.0125% *w/v*; 2—sunset yellow 0.025% *w/v*; 3—o-nitroaniline 0.0125% *w/v*; 4—allura red 0.0375% *w/v*; 5—azorubine—0.125% *w/v*; 6—1-aminoantraquionone 0.025% *w/v*; and 7—patent blue 0.125% *w/v*.

The distance between mobile phase entry position (from the main pump) and the mobile phase outlet (the detector cell tubings and the outlet electrode compartment) is longer than the distance between the sample application position (the sample pump tubing) and the outlet. For that reason, in the multichannel analysis we have obtained higher backpressure than in the single channel analysis as the total flow originated from the main pump only (except sample application time). We had to decrease the total flow of the mobile phase in OPLC to only 0.25 mL/min that, in any case, gave a backpressure 96 bar. However, as we have mentioned above, the electroosmotic effect can facilitate the mobile phase flow. Therefore, in HPLEC we were able to increase the total flow to 0.34 mL/min, with the backpressure 93 bar. The comparison of the performed multichannel analysis is presented in Figure 3. In the figure, there are only two overlaid (from all five) (electro)chromatograms obtained simultaneously (for clarity/readability reasons), with a 3 min delay of the sample application for the 2nd one in respect to the 1st. As can be seen, the 2nd (electro)chromatogram (grey) is more or less a reflection of the 1st one (black). The similarity (repeatability) is somewhat higher for OPLC than for HPLEC. This results from the fact that the baseline is less stable while using high voltage, probably due to gas bubbles formation and/or the influence of electroosmosis on the mobile phase flow. This may be a problem, especially while analyzing the solutes in concentration near to the detection limit. At a higher concentration of solutes, it is not a problem, as we have proven before [1] (in comparison, here the detector response is about 100 times lower). In any case, we believe that the stability of the baseline can be improved by the further separation of the mobile phase entry trough and the electrode compartment at the inlet side, to prevent the diffusion of gas being formed on the electrode to the separation system. This may be supported by the fact that in single-channel separation, where the mobile phase flowing through the separation channel originates from the sample pump and has no contact with the electrode compartment, this issue seems not to be a problem (Figure 2b,c). The further improvement of the HPLEC chamber cooling system may also produce some positive results. As our results show, the fluctuation of the baseline in the multi-channel HPLEC mode clearly affects the peak shape and the overall efficiency of separation, which are lower than in multi-channel OPLC and single-channel HPLEC. This mode of separation needs further improvement and optimization, especially at low concentrations of the sample. The procedure/parameters of multi-channel sample injection should be optimized because in both OPLC and HPLEC multi-channel separations presented, the peak width is higher and the efficiency of separation is lower than in the single-channel operational mode.

In our previous paper [1], we have proven that simultaneous, fully online, and fully automated multichannel analysis of simple drug samples are possible with our prototype equipment. Here, we show that more complex samples can also be analyzed using either the OPLC or HPLEC separation mode. This can be done using a relatively simple planar separation system, with standard, relatively cheap chromatographic plates. Moreover, it can provide high throughput and offer a wide range of variables/parameters that can be easily optimized to obtain the best separation. Yet, as discussed before, professional engineering and manufacturing are needed to reveal the true analytical potential of the equipment and the HPLEC technique.

### 2.3. Online Sample Injection with Offline Detection

**Figure 4:**
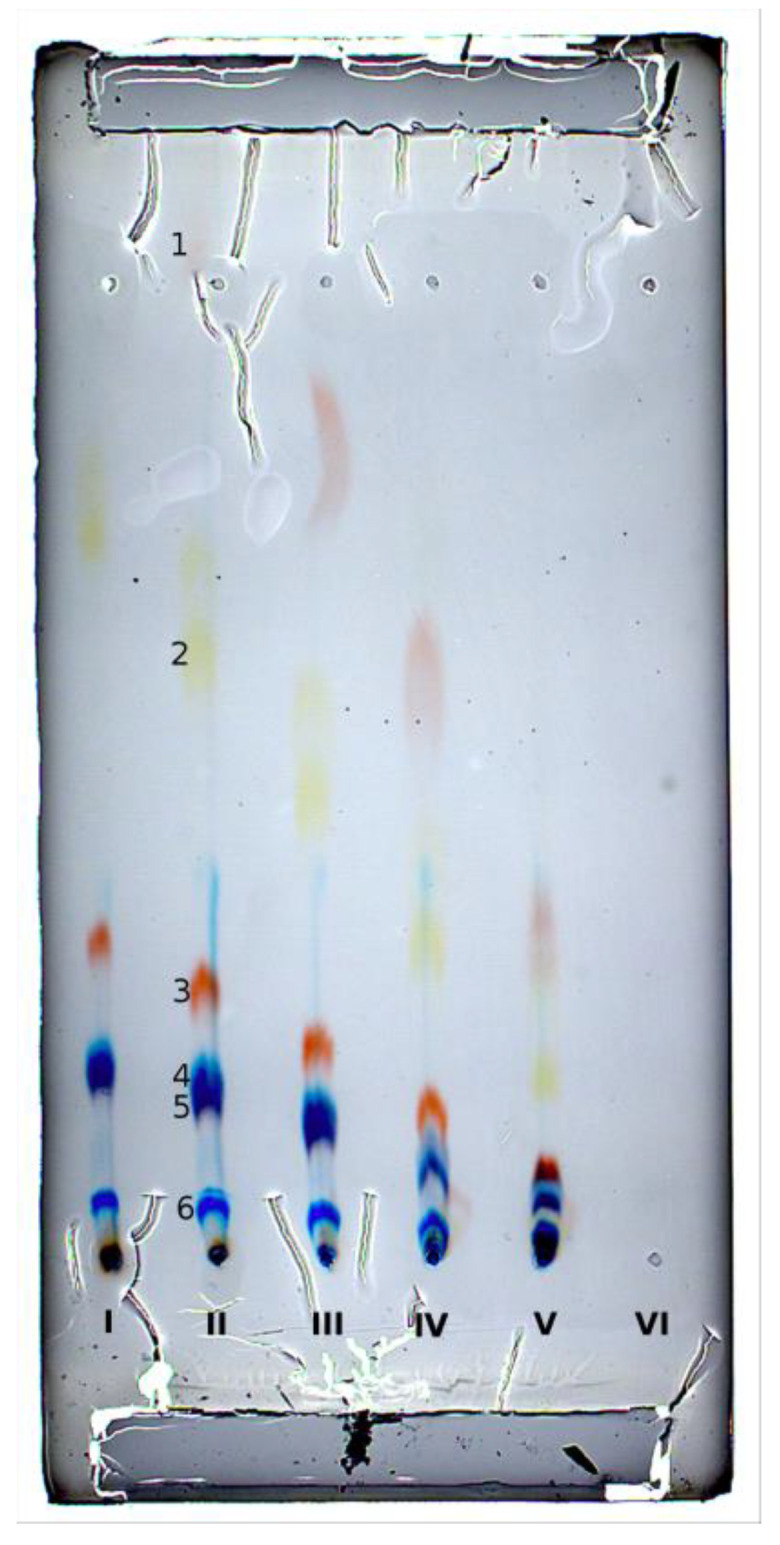
Chromatogram OPLC with online sample application and offline detection: 1 µL of the mixture was injected to the five separation channels, with 3 min delay between sample application in each subsequent channel. Separation system used: HPTLC RP-18 W chromatographic plates; water/methanol (3/2 *v/v*) with addition of 80 mM ammonium formate pH 3.0 as the mobile phase; total mobile phase flow 0.25 mL/min; voltage 0 kV; temperature 25 °C, cushion pressure 100 bar; and mobile phase backpressure 96 bar. Dye mixture: 1—ponceau red; 2—sunset yellow; 3—allura red; 4—brilliant blue; 5—azorubine; and 6—patent blue; concentration of each dye was 0.07% *w/v*.

The equipment presented was designed mainly for fully online, fully automated separations. In any case, its construction also enables offline sample application and detection. The example of OPLC with online sample application and offline detection is presented in Figure 4. The progressing separation of the dye mixture in 5 subsequent separation channels can be seen (6th channel of the selection valve was set to the waste). The delay of application between each subsequent channel was 1 min. The composition of the mixture was somewhat changed with respect to the one used in online experiments, to improve the visual detection. In the figure, it can be seen that the same solutes separated in subsequent channels lie on the straight line. This proves that the overall flow of the mobile phase and solutes is uniform throughout the whole adsorbent layer. Some adsorbent damage (and deflection of the ponceau red spot in channel 3) are visible. These arose during the release of the pressure from the pressure cushion and the removal of the plate from the HPLEC chamber. Probably a lower pressure, as used elsewhere [13,14], would be more suitable for offline detection. For this detection mode, a different flow ratio: sample/mobile phase was used than in online experiments, to obtain sample zone close to the round spot. Using the same ratio as in online separations resulted in the formation crescent sample zones. The pumping sample followed by the mobile phase through the sample inlet capillary results in radial chromatography (this effect still persists to some extent, despite the flow ratio change—see sample zone shape in Figure 4). This effect can be advantageous in some cases, e.g., with online detection, only the small central part of the crescent solute zone goes to the detector cell, producing a relatively narrow peak. However, as we have mentioned above, the sample application issue requires detailed investigation elsewhere. In any case, the HPLEC equipment presented here may work as well in both the offline and online detection mode.

### 2.4. Offline Sample Application with Online Detection

**Figure 5:**
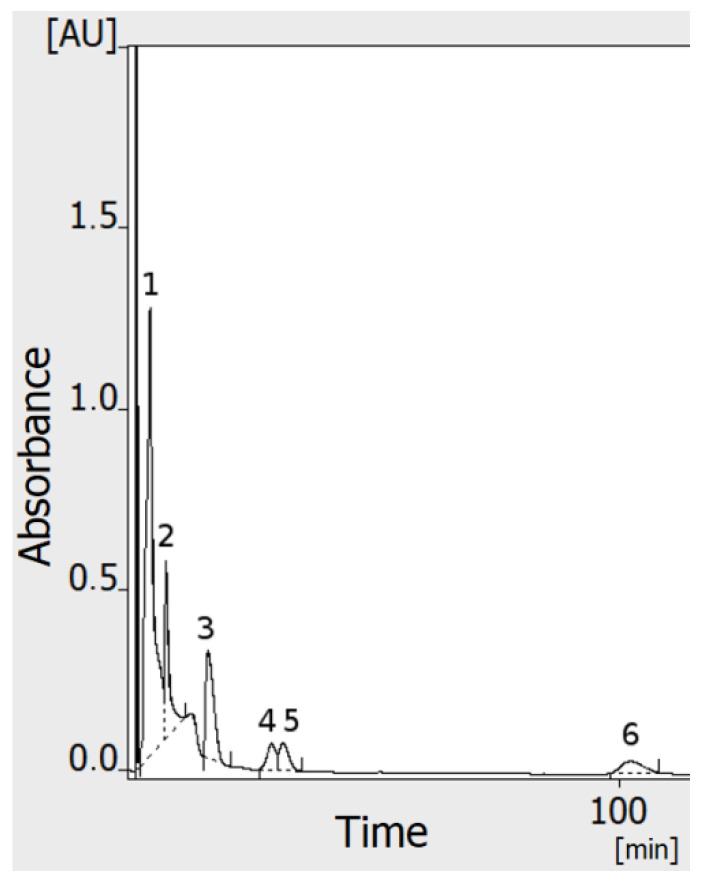
Chromatogram OPLC with offline sample application and online detection: 1 µL of the mixture was applied to the five separation channels using Linomat 5 aerosol sampler. Separation system used: HPTLC RP-18 W chromatographic plates; water/methanol (3/2 *v/v*) with addition of 80 mM ammonium formate pH 3.0 as the mobile phase; total mobile phase flow 0.25 mL/min; voltage 0 kV; temperature 25 °C, cushion pressure 100 bar; and mobile phase backpressure 96 bar. Dye mixture: 1—ponceau red; 2—sunset yellow; 3—allura red; 4—brilliant blue; 5—azorubine; and 6—patent blue; each dye concentration was 0.07% *w/v*.

The same dye mixture and the same separation system were used to demonstrate the offline sample application with the online detection mode, as seen in Figure 5. Only one of the six chromatograms obtained simultaneously is shown because, in contrast to the online sample-injection mode, here all the chromatograms overlap as there is no delay to the start of the analysis between subsequent separation channels. In general, the separation was successful and it proves that our HPLEC equipment can be used in the offline sample application mode. However, as can be seen in Figure 5, there is a significant baseline drift that interferes with the peaks. A similar drift was shown before for online detection in unequilibrated the OPLC systems [14,19]. The drift is also shown in Figure 1. It results from the separation system equilibration (adsorbent conditioning) process. It is clearly undesirable. As discussed above, the drift probably originates from impurities and/or from the air residue in the adsorbent layer. The proper washing of the chromatographic plate before use, with an acid or/and with the mobile phase [20], could possibly eliminate or at least limit the problem. Another potential solution (and probably the most effective) could be the purification of the adsorbent using the conditioning method described above—washing with the mobile phase, with the simultaneous application of high voltage. This issue requires further detailed investigation.

Both described experiments, concerning offline sample application or offline detection, were performed in the OPLC mode. In any case, high voltage can be applied at any time. It seems that there is no restriction in this matter in the online sample injection mode. In the offline sample application mode, with the unconditioned adsorbent layer, some setbacks can be encountered. As it has been discussed above, the presence of ionic impurities in the adsorbent may result in high electric current, which can even exceed the capabilities of the power supply. This, in turn, may result in extensive heating of the adsorbent layer, affecting the separation conditions. Consequently, the voltage that can be used in the analysis may be considerably restricted. Still, prior washing/purification, as mentioned above, could also be a solution to this problem.

The results show that our HPLEC equipment can be used in various separation (Table 1) and operational modes (Table 2). The proper analytical technique and method can be selected, depending on the needs and expectations. The independent optimization of many various operational parameters should enable one to obtain the best separation possible.

In our experiments, HPTLC RP-18W plates were used. This type of an adsorbent, due to the grain size (5–7 µm) and shape (irregular), may not be optimal for OPLC/HPLEC techniques as it may generate relatively high backpressure. To optimize the flow of the mobile phase and to reduce backpressure, specially dedicated adsorbents could be used. LiChrospher chromatographic plates from Merck, with spherical adsorbent particles, could be potentially useful in this matter. Unfortunately, it was not possible to buy these plates lately (the manufacturer reported some production problems/downtime).

## 3. Materials and Methods

### 3.1. Chemicals and Equipment

Certified analytical standard dyes were purchased from the Institute of Dyes and Organic Products (Zgierz, Poland). Ammonium formate and formic acid (both of an analytical purity grade) were purchased from Chempur (Piekary Śląskie, Poland). Methanol (for HPLC—super gradient) was purchased from POCH (Gliwice, Poland). Water used in all experiments was purified using HLP demineralizer from Hydrolab (Gdańsk, Poland). Glass-backed HPTLC RP-18 W plates were purchased from Merck (Darmstadt, Germany).

#### HPLEC Equipment

The prototype HPLEC equipment was designed and constructed in the Department of Physical Chemistry, Medical University of Lublin, and was presented and described in our previous paper [1]. The external pressure supply unit was ordered from P. W. Rafkop (Lubartów, Poland). The high-voltage power supply EV262 was bought from Consort (Turnhout, Belgium). Two quaternary HPLC pumps Azura P6.1L, the automatic six-channel selection valve Azura V2.1S, the autosampler Azura AS6.1L, and six UV detectors Azura UVD 2.1S (with analytical flow cells—path length 3 mm, capacity 2 µL) were bought from Knauer (Berlin, Germany). The circular thermostat AD07R-20 was bought from PolyScience, (Niles, IL, USA). The LINOMAT 5 semi-automatic TLC sampler was provided by CAMAG (Muttenz, Switzerland).

### 3.2. Dye Mixture

- For all online OPLC and HPLEC separations, a dye mixture of the following composition was used: 1—indigotine 0.0125% *w/v*; 2—sunset yellow 0.025% *w/v*; 3—o-nitroaniline 0.0125% *w/v*; 4—allura red 0.0375% *w/v*; 5—azorubine—0.125% *w/v*; 6—1-aminoantraquionone 0.025% *w/v*; and 7—patent blue 0.125% *w/v*.
- For offline sample application and offline sample detection experiments, another dye mixture was prepared (for better visual detection): 1—ponceau red; 2—sunset yellow; 3—allura red; 4—brilliant blue; 5—azorubine; and 6—patent blue; concentration of each dye was 0.07% *w/v*.

Dye mixtures were prepared by mixing and diluting 0.5% *w/v* dye stocks solutions. All stock solutions and final mixtures were prepared in water/methanol (1/1 *v/v*) solution.

### 3.3. Separation of Dye Mixtures

All experiments were performed at the temperature of 25 °C, applying 100 bar pressure to the cushion pressurizing the adsorbent layer. The mobile phase was the mixture of water/methanol (3/2 *v/v*) with the addition of 80 mM ammonium formate (final concentration), pH 3.0 (obtained by addition of formic acid to ammonium formate solution in water). Most of the HPLEC equipment modules were controlled by a computer with Clarity Chrom software (Knauer, Berlin, Germany). Only the high-voltage power supply, the pressure supply, and the circular thermostat were programed independently.

#### 3.3.1. Preparation of Equipment and Chromatographic Plate Conditioning

Before online experiments, the mobile phase was pumped through the adsorbent layer until the baseline was flat (at least for 1 h). The composition and flow of the mobile phase were the same as used in separation process. Before HPLEC separation, the voltage gradient was also applied: from 0 V to the final value used for separation, for 1 h.

For offline sample application and offline sample detection, chromatographic plates were not conditioned. Electrode compartments were filled manually before the separation.

#### 3.3.2. Sample Application/Injection

- For the online sample injection mode, using an autosampler, 1 µL of the sample solution was injected into the stream of the mobile phase pumped by the sample pump. Then, the sample solution in the stream of the mobile phase was delivered to the virtual channel of the chromatographic plate.
- For all fully online (sample application and detection) experiments, the ratio of the flow velocity from the sample pump to the total flow of the mobile phase was 15%. For experiments with offline detection, this ratio was 4%. For the offline sample application mode, only the main mobile phase pump was used.
- For single-channel experiments, the flow of the mobile phase provided by the sample pump (after sample application) was sustained during the whole experiment.
- For multi-channel online experiments, the flow from the sample pump was applied sequentially to one channel for 3 min and then switched to the next channel; hence, there was a 3 min delay in sample injection between the subsequent channels. After injection of the samples into the five channels, the mobile phase pumped by the sample pump was switched to the waste and the sample pump was stopped. Only five of the six channels were used (no. 1–5) as position no. 6 of the selection valve was used to switch the flow from the sample pump to the waste before and after the sample injection.
- For offline detection experiments, the procedure was similar, but the flow from the sample pump was applied for 1 min. There was only 1 min delay between consecutive sample injections for each subsequent channel.
- For the offline sample application mode, 1 µL of the sample solution was applied for each separation channel, and 40 mm from the lower edge of the chromatographic plate (behind the point of the sample inlet, for the online application mode) as a 4 mm band. The aerosol applicator Linomat 5 was used at a sample application velocity of 70 nL/s.

#### 3.3.3. Separation Conditions

- For all OPLC experiments, the electrode compartment at the mobile phase inlet side was closed (the compartment was not rinsed) so that the real flow of the mobile phase through the adsorbent layer was equal to the total flow set to the HPLC pumps. At the outlet side, the flow was split between flow cells of detectors: 8.4% of flow for each cell, and the outlet electrode rinsing/venting valve was 50.4%.
- For HPLEC experiments, the venting valve of the inlet side electrode compartment was opened to facilitate the constant rinsing of the electrode with the mobile phase. The flow through the venting valve was restricted to 2% of the total mobile phase flow. On the outlet side, the flow was 7% for each detector cell and 56% for the electrode venting valve.
- For all single-channel analysis, the total flow of the mobile phase was 0.33 mL/min. The backpressure at the inlet depended on the voltage applied and was equal: 93 bar for 0 kV, 82 bar for 3.7 kV, and 80 bar for 3.8 kV.
- For multi-channel experiments at 0 kV, the total flow of the mobile phase was 0.25 mL/min and the backpressure 96 bar. At 4.2 kV, the total flow was 0.34 mL/min and the backpressure 93 bar.

#### 3.3.4. Solute Detection and Identification

- In online detection experiments, all solutes were detected at 256 nm wavelength, simultaneously using six independent flow cells and detectors, each collecting samples/eluents from six independent separation channels of the HPLEC chamber. Six independent signals were recorded and overlaid in a single analysis (chosen signals may be shown/hidden at any time). A sampling rate was 2 Hz, and the time constant was 1 s. The migration time was established automatically by the software. The identification of solutes was based on the comparison between the migration time of the mixture components and the single standards separated under the same conditions.
- For offline detection, the identification of solutes was performed by the visual comparison of colors and the migration distance (sequence) of the spots, as well as by comparing the data obtained in online experiments.

## 4. Conclusions

Our new HPLEC equipment was designed to enable separation in many different modes, including OPLC, PPEC, HPLEC, and online or offline sample application and detection. It allows for independent optimization of various operational parameters, such as working pressure, temperature, mobile phase flow and flow ratio (sample/mobile phase), flow splitting, and voltage. The main purpose of our equipment is the high-throughput, multichannel, fully automated online separation of many samples at the same time. The early results presented here prove that these theoretical assumptions were fulfilled. The equipment met our expectations and can be successfully used for singe- and multichannel HPLEC separations.

The results confirm that HPLEC combines the advantages of column/capillary (high performance/throughput, automation, and separation system equilibration) and planar separation techniques (the simultaneous separation of multiple samples, as well as the numerous possibilities of sample application and detection, and derivatization—in offline mode), while overcoming their limitations, as suggested before [1]. Moreover, it combines the advantages and overcome the drawbacks of OPLC and PPEC. The mobile phase flow does not depend on the electroosmotic flow and can be optimized independently. The mobile phase composition can be optimized to obtain the required retention, without considering its influence on the mobile phase flow. The voltage can be used to change the separation selectivity and/or to facilitate the mobile phase flow and decrease the backpressure. Both approaches may be used to speed up the analysis. The mobile phase flow to voltage ratio is crucial for the selectivity of HPLEC separation. It must be taken into account while transferring the separation conditions between systems with continuous and discontinuous flow from the sample pump.

Online sample application remains a challenge. It requires the investigation and optimization of operational parameters, and possibly some technical improvements. The ionic impurities of the adsorbent layer may be a problem, especially in the offline sample application mode. In the online mode, the equilibration of the separation system may be beneficial and recommended. These issues require further investigation and further solutions. Moreover, professional engineering and manufacturing is needed to reveal the true analytical potential of HPLEC.

## Figures and Tables

**Table 1:** Comparison of OPLC and HPLEC techniques.

OPLC	HPLEC
No voltage applied	High voltage applied
Separation selectivity based on solute retention	Separation selectivity based on solute retention and electrophoretic mobility
Rapid mobile phase flow	Even more rapid mobile phase flow (lower backpressure)
Mobile phase flow generated by pressure gradient	Mobile phase flow generated by pressure gradient and amplified by electric field
Stable baseline	Less stable baseline
Single- or multichannel separation possible	Single- or multichannel separation possible
Online or offline sample application	Online or offline sample application
Online and/or offline sample detection	Online and/or offline sample detection

**Table 2:** Variants of conducting the analysis with the HPLEC equipment and their features.

Fully Online Analysis	Offline Sample Application and Online Sample Detection	Online Sample Application and Offline Sample Detection	Offline Sample Application and Offline Sample Detection
The entire analysis is carried out on one computer-controlled device	Sample application as an additional operation excluded from online process	Sample detection as an additional operation excluded from online process	Sample application and detection excluded from separation process
Full automation	No full automation; partial manual operation required	No full automation; partial manual operation required	Automation restricted
Separation in fully equilibrated system	Separation system not equilibrated	Separation in fully equilibrated system	Separation system not equilibrated
Stable baseline	Drifting baseline	No influence of mobile-phase composition on baseline shape (visual or densitometric detection)	No influence of mobile phase composition on baseline shape (visual or densitometric detection)

## Data Availability

Not applicable.

## Sample Availability

All samples and other chemicals are commercially available.
